# Institutional Treatment of Fevers

**Published:** 1903-08-22

**Authors:** 


### INSTITUTIONAL TREATMENT OF FEVERS.

The medical supplement to the Metropolitan Asylums Board's Annual Report is always an interesting document containing as it does statistics compiled from a large mass of material and showing at a glance what is the present position of medical science with regard to the class of patients who come under the Board's care. Among other interesting features in the recently-issued report for 1902 are statistical tables dealing will small-pox, laparotomy for perforated typhoid ulcer, tracheotomy and intubation, complications of scarlet fever, diphtheria and enteric fever. During the year 7,961 cases of small-pox were admitted into the Asylums Board Hospitals, and of these 16·6 per cent. terminated fatally. Of 9,659 cases admitted during the years 1901 and 1902, 6,945 had been vaccinated, the mortality amongst whom was only

7·34 per cent. In 436 cases it was doubtful whether vaccination had been performed; amongst these cases the mortality rate was 39·22, while of the 2,278 who were admittedly unvaccinated the mortality was at the rate of 33·06 per cent. During the epidemic there were, under 10 years of age, 134 vaccinated cases with two deaths, a mortality of 1·49 per cent., while there were 1,274 unvaccinated cases in children under 10 years of age, of whom 442 died—a percentage mortality of 34·7. The tables showing the results of laparotomy for perforation of the intestine in enteric fever are perhaps a little disappointing. Twenty-two such cases were submitted to operation and of these only two recovered; the mortality for these 22 cases thus working out at 90·9 per cent. Still, the two recoveries must be looked upon as two lives saved, since spontaneous recovery is so rare. In both successful cases the perforation had occurred late in the disease, on the forty-ninth day in one (on the seventeenth day of a relapse), and on the forty-third day in the other. In two of the 22 cases autopsy revealed two perforations, and in one of these, in which the complication occurred on the twenty-fourth day of illness in a man 23 years of age, the two perforations were situate in the large bowel, one being in the rectum, 8 inches from the anus, and the other in the sigmoid flexure, 6 inches higher up. There were also present numerous shallow ulcers in the large intestine with but little ulceration in the ileum. The tables by which the results of tracheotomy and intubation may be compared show in cases of primary diphtheria a mortality of 32·5 per cent. for tracheotomy and 34·2 per cent. for intubation. One might perhaps infer that the balance in favour of tracheotomy is really greater than appears from these figures since it is likely that some of the more severe cases were submitted to tracheotomy. The total mortality for diphtheria was 11·0 per cent., and here again one cannot but be a little disappointed. Although the introduction of the antitoxin treatment has reduced the case mortality of diphtheria in the fever hospitals from over 30 per cent., at which figure it stood a few years ago, yet it cannot be claimed that the present figure is as low as it should be in view of the fact that if the serum be administered on the first day of the disease a fatal termination is almost invariably prevented. The responsibility rests, of course, not with the medical officers attached to the fever hospitals, but with the patients' parents or guardians and the general practitioners into whose hands the cases are first brought, and upon whose diagnostic acumen and judgment the issue of the disease therefore chiefly depends. An important point to which we see no reference is the manner in which the antitoxin is administered. If it be found in the light of extensive experience that the serum can be efficiently administered by rectal injection, it is probable that its use will be resorted to earlier in any given case, and more frequently in cases where there is some doubt about the diagnosis, with the result that a further considerable reduction in the mortality from diphtheria may occur. However this may be medical men in general practice should be sufficiently awake to the fact that a single day's delay in resorting to antitoxin treatment in a case of diphtheria may, and frequently does, cost the patient's life. The tables showing the complications of scarlet fever, diphtheria and enteric are all of interest. In the case of diphtheria albuminuria was noticed in 32·98 per cent., while nephritis only occurred in 0·49 per cent. Paralysis was met with in 17·07 per cent., otitis in 6·06 per cent. Scarlet fever as a complication occurred in 5·33 per cent. of cases. The danger of this latter complication, as also of post-scarlatinal diphtheria is one drawback to the institutional treatment of these affections. Attempts to surmount the difficulty by having diphtheria and scarlet fever cases accommodated in different hospitals have been carried out but were not successful in bringing about a reduction of the cases.

